# ‘They can induce and exacerbate each other’ – the complex interplay between domestic abuse and the perimenopause: a qualitative study with female survivors

**DOI:** 10.1186/s12905-025-04080-9

**Published:** 2025-11-29

**Authors:** Claire Mann, Kathryn Hinsliff-Smith, Sally Olewe-Richards

**Affiliations:** 1 CHILL (Centre for Health Innovation, Leadership & Learning), NUBS University of Nottingham, Nottingham, UK; 2 Health & Life Sciences De Montfort University, Leicester, UK

**Keywords:** Domestic abuse, Domestic violence, Menopause, Intimate partner violence, Mid-life, Women’s health

## Abstract

**Background:**

Domestic abuse (DA) and perimenopause are each known to profoundly impact women’s health, yet their intersection remains largely unexplored. This study reveals how these experiences collide to create unique vulnerabilities and unexpected opportunities for transformation. This qualitative study explores how DA survivors experience perimenopause, examining the complexity and support needs that emerge when these experiences overlap.

**Methods:**

Fifteen DA survivors participated in focus groups (and one individual interview) facilitated by a community leader of a DA survivors group exploring their perimenopause experiences. Data were analysed thematically using the one sheet of paper (OSAP) technique, with a DA survivor community leader (third author) involved throughout to support ethical engagement and participant well-being. Analysis revealed how trauma and hormonal changes interweave to shape help-seeking and survival.

**Results:**

Three interconnected themes emerged: (1) symptom confusion and overlapping conditions - participants struggled to distinguish between trauma responses, hormonal changes, and pre-existing conditions; (2) weaponisation and empowerment - perpetrators exploited perimenopausal symptoms for coercive control, yet paradoxically, hormonal changes sometimes catalysed women’s decisions to leave; (3) barriers and facilitators - system failures drove survivors toward peer support networks that provided validation unavailable from professional services. Participants described heightened anxiety, mood changes, and sleep disturbances intensified by current or past abuse. The weaponisation of perimenopausal symptoms represents a previously unreported form of coercive control. Conversely, some participants described perimenopause as a moment of emotional clarity, contributing to decisions to leave relationships with an abusive partner.

**Conclusions:**

The intersection of DA and perimenopause creates unique vulnerabilities that current UK support systems fail to address. Healthcare providers require training to recognise how trauma and hormonal symptoms can mask or mimic each other. The finding that perimenopause can serve as both a tool of abuse and a catalyst for liberation challenges deficit-focused narratives around both DA and perimenopause. Integrated, trauma-informed approaches are urgently needed across healthcare and support services.

**Supplementary Information:**

The online version contains supplementary material available at 10.1186/s12905-025-04080-9.

## Background

Awareness of menopause in the United Kingdom (UK) has increased significantly, driven by greater visibility in mainstream media and advocacy efforts [1–3]. Perimenopause, which we focus on in this paper, is defined by NICE as the presence of vasomotor symptoms that have recently started and any changes in menstrual cycle [4]. However, the symptom experience is much broader, encompassing physical symptoms such as hot flushes, night sweats, joint aches, and fatigue; psychological symptoms including mood changes, anxiety, and irritability; and cognitive symptoms like brain fog and memory difficulties. Its duration varies, but it typically lasts a few years and leads to menopause. Menopause is defined as 12 months after the last menstrual period [4]. Perimenopause typically starts in the mid-40s, although it can be earlier, and its duration varies, typically lasting several years before culminating in menopause. Menopause is defined by NICE as not having had a period for at least 12 months (in those not using hormonal contraception) [4], with the average age of menopause in the UK being 51 years. There is a growing recognition for a wider understanding of perimenopause symptoms and their impact on women’s lives, including wider use and prescribing of hormone replacement therapy (HRT) nationally [5–7].

However, whilst this shift in awareness is welcomed, it has highlighted significant disparities in access to menopause treatment and support, particularly amongst disadvantaged populations. These disparities reflect broader systemic health inequalities and underscore the need for more equitable approaches to menopause care. It is important to note that not all women experience natural menopause. Some women undergo surgical menopause following hysterectomy with the removal of the ovaries, often due to conditions such as endometriosis, fibroids, or gynaecological cancers. Others experience chemical menopause through treatments such as chemotherapy or hormone therapy for breast cancer. These induced menopauses typically result in more abrupt and severe symptoms due to the sudden rather than gradual decline in hormones, adding another layer of complexity for women who may also be navigating trauma from both their medical experiences and any concurrent DA.

Domestic abuse (DA) is defined as ‘an incident or pattern of incidents of controlling, coercive, threatening, degrading and violent behaviour, including sexual violence, in the majority of cases by a partner or ex-partner [8] There is an absence of reliable prevalence data on DA in the UK[8]. The Crime Survey of England and Wales (CSEW) provides the most comprehensive recorded information available. The most recent data, for 2023, estimated 1.6 million women experienced DA[9]. National referral estimates suggest that less than one in ten women who experience DA receive support from a refuge or community-based support service [10]. This highlights significant gaps in service provision and suggests that many victims are not captured in official statistics, either because they do not reach out for help or because services are unable to provide support when they do.

Recent DA research in the UK has focused primarily on different forms of DA, including coercive control and gendered experiences of justice [11, 12] and mental health impacts [13, 14]. The psychological impact of DA, including anxiety, depression, and PTSD, has also been well-documented [15, 16] whilst research has explored how social inequalities can shape abuse experiences [17]. Studies highlight how coercive control can persist post-separation through financial abuse, stalking, and extended family court processes. Rather than following a linear timeline, DA and menopause can intersect in multiple ways - some women experience current abuse during perimenopause, while others manage trauma responses from past abuse that may be triggered or intensified during this hormonal transition [11, 12]. The relationship between DA and menopause is complex, as women may experience abuse at any life stage - before, during, or after their menopausal transition. Some women may be experiencing current abuse during perimenopause, while others may be dealing with trauma responses from past abuse that can be triggered or intensified by hormonal changes. Additionally, coercive control and other forms of DA can persist across decades, meaning some women face both ongoing abuse and menopausal symptoms simultaneously. This temporal complexity means that DA-related trauma symptoms and perimenopausal symptoms may co-occur regardless of when the abuse originally occurred.

Survivors of DA can experience complex, wide-ranging physical and mental health symptoms that persist long-term due to the trauma they experience. Similarly, menopause involves multiple phases with symptoms that significantly impact women’s physical and mental well-being. However, there is limited research exploring how these experiences intersect for DA survivors and the consequential impact on their support experiences.

One recent international research study has begun to reveal connections between DA and menopausal experiences [18]. This study demonstrated that experiencing (partner) violence increased the risk of early menopause, worsened menopausal symptoms, and resulted in a lower quality of life. Further targeted research exploring this specific relationship remains relatively scarce, presenting a significant gap in knowledge for women’s health. Women with histories of DA report increased menopausal symptoms, including sleep difficulties, vasomotor disturbances, and vaginal issues [19]. These symptoms are particularly pronounced among women with Complex Post-Traumatic Stress Disorder (CPTSD) symptoms, which commonly affects DA survivors, suggesting a potential compounding effect of trauma during perimenopause, adversely affecting physical, sexual, and psychological health [18, 20]. Recent work in the USA [21] explored emotional abuse and linkages to reported increases in menopausal distress, including higher incidences of night sweats and painful intercourse. This study also found that women with experiences of DA alongside PTSD histories reported higher instances of menopausal symptoms, complicating diagnosis and support provision.

Such overlaps between the symptoms can make it difficult for healthcare professionals to distinguish between trauma-related symptoms and those associated with perimenopause, potentially leading to misdiagnosis, delayed treatment, or inappropriate support. This has the potential to lead to inadequate or inappropriate treatment options. Existing research has identified multiple barriers throughout the symptom management pathway for menopausal women [21]. These range from lack of empowerment to seeking medical advice to healthcare professionals’ dismissal of symptoms or failure to recognise less common menopausal presentations. The consequences extend beyond individual health outcomes. For DA survivors experiencing perimenopause, these challenges may be compounded by the complex interplay of trauma responses and hormonal changes, creating additional layers of diagnostic uncertainty. When these experiences intersect, women may face a ‘double burden’ of symptoms that are poorly understood by healthcare providers and inadequately addressed by existing support systems. This intersection is particularly concerning given that experiencing abuse at any point in a woman’s life can have detrimental impacts on her health that may be acute during mid-life and menopausal stages [17]. 

Despite the clear potential for these experiences to intersect and create unique challenges, there remains a significant gap in understanding how DA survivors navigate perimenopause and access appropriate healthcare support.

This paper aims to explore how the overlap between DA experiences and perimenopausal symptoms affects women’s healthcare-seeking experiences, particularly focusing on the challenges of symptom recognition and accessing appropriate support.

## Methods

### Design

The data reported here forms part of a wider qualitative study exploring diverse views on menopause, digital health and artificial intelligence (AI) which is being used to explore patterns in health experiences.

We recruited a large number of participants experiencing health inequalities, generating significant qualitative data. We conducted 3 separate analyses; the first is in relation to data about digital health and the second reporting on the findings in relation to women experiencing health inequalities which are reported elsewhere [citations]. This current manuscript reports on the data and analysis from participants (women) experiencing DA.

We utilised a social constructivist worldview with a goal to understand lived experience and co-create knowledge through interactions with participants. Analysis for this paper takes a pragmatic approach to understanding the gap in women’s healthcare delivery, where DA and perimenopause can cause mistreatment. We understand that DA and perimenopause are understood not as fixed phenomena, but as experienced differently by each participant. We therefore take a relativist ontological position, assuming that multiple realities exist and are shaped by individual experiences and social contexts. This view supports the use of qualitative methods that allow for rich, in-depth exploration of diverse perspectives. Through this approach, knowledge is constructed through conversations between participants and with the researcher. This subjectivist epistemological position contributed to our decision to take a partnered approach to the work.

A partnered approach involves collaboration with stakeholders throughout the research process and fosters shared ownership and mutual benefit [22]. Our semi-structured interview guide was designed by our lead researcher (CM) and the community leader (SOR) to explore several key areas, including managing and identifying symptoms, impact of symptoms, accessing help and health interventions. The questions were limited to allow free speech on the topic for a period of up to one hour per group. The questions were homogenised to be used by other groups of women who experience health inequalities, and as such, there are no specific questions related to DA. All data related to DA was emergent, emerging from women’s personal experiences.

The focus group interviews were organised and facilitated by a community leader and expert in DA and menopause (SOR), who is a co-author on this paper. The research design prioritised participant safety and well-being through the involvement of a trusted community leader as facilitator, creating an environment where DA survivors felt secure in sharing their experiences.

### Participants & setting

The study aimed to recruit 15 participants across 3–5 focus groups to discuss their experience of menopause and DA. We used focus groups to encourage discussion about this taboo topic area since previous research has shown that people may be more, rather than less, likely to self-disclose or share personal experiences in group rather than dyadic settings [23]. From a feminist perspective, focus groups can facilitate, rather than inhibit, discussion and people can feel relatively empowered and supported in a group situation, surrounded by their peers or friends. They may also be more likely to share experiences and feelings in the presence of people whom they perceive to be like themselves in some way [23, 24]. 

We aimed to recruit 15 women, and this was based on several previous studies that suggest data saturation might be reached at this number of participants [25, 26]. This was also for pragmatic reasons of timing, given for the project conclusion. The study was advertised by an online post to women who were members of a national DA support group by their community leader (SOR). Inclusion criteria were women aged 40–65 with current or historic experience of menopause and current or historic experience of DA. Women who expressed interest were sent the information pack and consent form and invited to share their availability for an online group discussion. Groups were organised by SOR according to participant availability to take place online using Microsoft Teams©. Participants had familiarity with the community leader (SOR), facilitating honest and open communication on this taboo topic. Co-producing the work with a community leader involved throughout the research process ensured ethical engagement and participant well-being.

A detailed interview schedule was co-designed with community partners. All potential participants were provided with study information, provided individual consent and were provided with a voucher for £25 as a convenience allowance in accordance with UK NIHR recommended rates.

### Data collection

Data were collected by the community researcher (SOR) between May and July 2024. We recruited participants to 5 focus groups, but one participant was unable to attend the final focus group and requested an individual interview as an alternative. We recognise the complication of different data types but felt it important not to exclude a potential participant.

We therefore conducted four online focus groups, each with three participants, one with two participants and one individual interview. Each interview lasted between fifty and ninety minutes. The online format, whilst presenting some challenges with technology, was a positive mechanism for inclusion in the research process. Using online group interviews as our method enabled the inclusion of participants nationally and provided all with a level of control over their environment during the interviews, potentially enabling more open dialogue about sensitive topics [23–26] All interviews were digitally audio recorded, transcribed verbatim and anonymised by the lead researcher (CM) and checked by the community researcher (SOR). For transparency, all participants were asked how they would prefer to receive the research findings for review and were sent a copy of the finalised digital report and, invited to a report launch event in September 2024 to discuss the relevance and applicability of the findings. Participants expressed feeling empowered by this co-creation process.

### Data analysis

A deductive approach was adopted for the data analysis, guided by predefined conceptual areas informed by the study’s research questions. To ensure depth and rigour, initial theme identification was carried out by two researchers (CM, SOR), who employed Ziebland and McPherson’s One Sheet of Paper (OSOP) technique [27]. This method involved independently distilling the core insights from each transcript onto a single sheet, allowing each researcher to visualise key patterns and contradictions within the data. The OSOP technique facilitated concise but comprehensive mapping of participants’ narratives, enabling iterative comparison and synthesis through discussion. To enhance intercoder reliability, the research team engaged collaboratively in the coding process involving regular discussions and cross-checking of coded data. This allowed for the integration of analytical perspectives from our diverse community research team, helping to refine coding categories and ensure consistency in interpretation across researchers.

Building on these initial summaries, the full research team (CM, KHS, SOR) undertook a discursive analysis approach. This second-level analysis was characterised by collaborative and reflexive discussions, which allowed for deeper exploration of emerging themes and sub-themes. Through this iterative process, the team refined and expanded the thematic framework, ensuring it captured both the complexity and nuance of participants’ lived experiences. The layered analytical strategy enabled a richer understanding of the intersections between DA and menopausal experience.

In the next section, we present the results and our interpretation of key themes along with exemplary quotes.

## Results

Overall, our findings revealed three interconnected themes that illuminate women’s experiences at the intersection of DA and perimenopause: (1) Symptom confusion and overlapping conditions, (2) Weaponisation and empowerment, and (3) Barriers and facilitators. These themes reveal a progression from individual confusion through relationship dynamics to system-level responses, demonstrating how the intersection of DA and perimenopause creates cascading challenges that affect every aspect of survivors’ help-seeking experiences.

### Participant characteristics

The 15 participants were aged between 40 and early 60 s, had or were experiencing perimenopause symptoms and had experienced DA. The participants were recruited from across various geographical locations in England; 14 were employed and 13 were mothers. The sample represented diversity across ethnicity, employment status, parental status, and sexual orientation. Demographically, the majority (12) of participants were white, but we also had participants representing diverse cultural groups, including Pakistani (1), Black (1) and mixed heritage (1). Age UK reports that 1 in 5 women over 50 belongs to an ethnic minority group, and so our participants are broadly proportionately representative of the UK population [28]. 

### Theme 1: symptom confusion and overlapping conditions

Participants described a complex web of uncertainty when trying to understand the origins of their symptoms. This confusion was compounded by limited knowledge about perimenopause, existing health conditions, and the ongoing effects of trauma from their DA experiences. The inability to distinguish between trauma responses and hormonal changes created a diagnostic maze that left many women feeling lost and overwhelmed.

#### Menopause knowledge and awareness

Many participants had limited knowledge about perimenopause, which contributed to their uncertainty about symptom origins. Cultural factors played a significant role:

*“I come from Africa and this kind of stuff is not normally discussed*,* so I’m not really 100% sure about a lot to do with menopause”* (Participant 4).

#### Symptom origin uncertainty

Participants consistently struggled to distinguish between symptoms caused by hormonal changes, stress from abusive relationships, or other life circumstances. This created significant anxiety:

*“In the last few months*,* I’ve been losing my hair*,* and yeah*,* that’s probably part of menopause or stress. So*,* this is what I do*,* I juggle*,* is it that or is it that?”* (Participant 7).

The diagnostic uncertainty was particularly acute for those still processing recent abuse:

*“It was really hard to pinpoint which was the perimenopause and what was also just an effect of being in my house at that point in time”* (Participant 3).

One participant’s experience powerfully illustrates how abusers can exploit this uncertainty. She sought medical help thinking she had Alzheimer’s, only to later realise:

*“I went to the GP… where I thought I was going mad… he [abusive partner] was telling me I’ve got mental health problems […] and now I can look back at it and it was him gaslighting me”* (Participant 3).

#### Comorbidities and complexity

The presence of existing health conditions further complicated symptom attribution. Participants reported various conditions, including fibromyalgia, endometriosis, polycystic ovary syndrome, attention-deficit/hyperactivity disorder, depression, post-traumatic stress disorder and bipolar disorder. This complexity created an overwhelming diagnostic puzzle:

*“Sometimes it’s really hard to figure out… which things are actually down to menopause and how many things are cross-cutting… I don’t actually know*,* and I haven’t got the time or the energy to monitor”* (Participant 9).

This foundational uncertainty about symptom origins sets the stage for how these experiences play out in relationships and healthcare settings, creating vulnerabilities that can be exploited while also opening unexpected pathways to clarity.

### Theme 2: weaponisation and empowerment

Building on the confusion described in Theme 1, participants’ experiences revealed how perimenopause and abusive relationships interact in paradoxical ways. Whilst perpetrators weaponised symptoms as tools of control, the hormonal transition sometimes provided unexpected clarity and strength that facilitated escape from abuse.

#### Weaponisation of symptoms

Several participants described how abusers exploited perimenopausal symptoms for psychological abuse. This included gaslighting about symptoms:

*“They’re capable of telling you stuff that actually hasn’t happened as well to make you think you’re going mad”* (Participant 12).

Perpetrators also undermined survivors’ autonomy by controlling treatment decisions:

*“He was telling me that I’ve got issues and if I wasn’t going to go to the doctor I needed to try and maybe sort it out homoeopathically”* (Participant 12).

Control extended to bodily autonomy, with one participant describing how her medical decision sparked abuse:

*“My GP suggested I had the coil fitted and that was then actually a catalyst to my ex wondering why on Earth would I have a coil fitted because we weren’t having sex”* (Participant 2).

#### Menopause as catalyst for change

Remarkably, several participants described how perimenopause provided emotional clarity and reduced tolerance for abuse:

*“All the people pleasing tendency part of me would reach a peak at the point when I was really hormonal and it was just like*,* ‘No*,* I’m just not taking this shit anymore’… I still feel like there’s an element of rebirth”* (Participant 8).

This clarity often coincided with decisions to leave:

*“Bizarrely as my menopause kicked in*,* that’s when I got to the point where I was leaving. I’d had enough”* (Participant 6).

For some, hormone replacement therapy combined with freedom from abuse proved life-saving:

*“If I was in that abusive relationship still*,* and not treated for perimenopause with my HRT*,* I don’t think I’d have survived”* (Participant 2).

#### Family and relationship impacts

The bidirectional relationship between abuse and symptoms created vicious cycles. Abuse worsened symptoms:

*“Everything about this*,* any symptoms related to anxiety or depression*,* especially irritability and feeling really low*,* increased being around him always”* (Participant 5).

Many participants recognised this interconnection:

*“They can induce or exacerbate one and each other”* (Participant 6).

The intersection of perimenopause and abuse also manifested in intimate relationships, with participants describing confusion about whether loss of libido was hormonal or a response to abuse, and experiences of sexual coercion during vulnerable states.

This theme demonstrates how the very symptoms that create vulnerability can also spark transformation, leading directly to the challenges survivors face when seeking help from systems ill-equipped to understand this complexity.

### Theme 3: barriers and facilitators to healthcare seeking

The diagnostic uncertainty and relationship dynamics described in the previous themes culminated in significant barriers when participants sought healthcare and support. The intersection of abuse experiences and hormonal changes created unique challenges that existing healthcare systems often failed to recognise or address adequately.

#### Relationship impact on symptom uncertainties

The context of abusive relationships prevented many from prioritising their health:

*“There were bigger things going on… I didn’t suffer like most women suffered with the menopause*,* but come to think of it*,* it’s because I put it back in second place importance”* (Participant 13).

#### Healthcare provider responses

Experiences with healthcare providers varied dramatically. Whilst some found support:

*“My GP… did listen to me and allowed me to make choices for myself”* (Participant 14).

Others encountered providers who failed to recognise the complex intersection of abuse and perimenopause:

*“Concurrently I was in a domestic abuse relationship and when I went to the GP*,* I was basically just put on antidepressants”* (Participant 6).

#### Low self-esteem negating access seeking

Trauma significantly impacted participants’ ability to advocate for themselves:

*“Because of my lack of self-esteem and self-worth*,* I didn’t feel like I could chime up. So*,* I waited the three months”* (Participant 14).

#### Alternative support seeking

When formal healthcare failed, participants turned to other resources, though with mixed results:

*“I bought books and accessed as much information as I could on the internet… My GPs weren’t ever so helpful”* (Participant 12).

Financial barriers, often resulting from economic abuse, limited options:

*“It’s all-consuming in your head because you’re worrying about the stupidest things… money does mean everything now”* (Participant 11).

#### Peer support as a lifeline

Despite formal system failures, peer support emerged as invaluable:

*“My main source is actually being in conversations with other women… It’s always helpful to talk to women who are going through the same symptoms”* (Participant 5).

#### Self-care and survival mode

Many participants described existing in “survival mode”, where basic functioning took precedence:

*“I just think what the hell do I even care? Because you get like that*,* you’re like ‘Well I’m alive and it’s fine and it’s OK’”* (Participant 13).

This theme explores some of the key barriers and facilitators to healthcare services in this population. It demonstrates the impact of a DA relationship and perimenopause on access and healthcare provider responses. We outline how low self-esteem negates access seeking and how women seek alternative support, including peer support as a lifeline and how women approach self-care in survival model.

## Discussion

This qualitative study aimed to explore the experiences of 15 UK women navigating perimenopause whilst recovering from DA, and to understand how these experiences impact their health and help-seeking. Three interconnected themes emerged: symptom confusion and overlapping conditions, where women struggled to distinguish between trauma responses and hormonal changes; weaponisation and empowerment, revealing how perpetrators exploit perimenopausal symptoms whilst the transition can paradoxically catalyse escape; and barriers and facilitators, highlighting how system failures drive survivors toward peer networks.

This is the first UK study to examine how DA and perimenopause interact to create unique challenges for healthcare access, extending existing knowledge about both phenomena whilst revealing novel insights about their intersection. Our findings both support and extend existing literature on trauma and menopause. Previous research has documented increased menopausal symptoms among DA survivors [19, 21]. Our study reveals the mechanisms through which these intersections create compound vulnerabilities. The symptom uncertainty experienced by participants extends beyond simple symptom overlap, reflecting a complex interplay where trauma histories, cultural silences around menopause, and existing health conditions create what participants described as an overwhelming diagnostic puzzle.

This diagnostic complexity has profound implications for healthcare provision. When women cannot determine whether symptoms stem from hormonal changes, trauma responses, or pre-existing conditions, help-seeking becomes fraught with uncertainty. Healthcare providers, often lacking training in both trauma-informed care and menopause management, may default to simplistic solutions – demonstrated by participants who were prescribed antidepressants or contraceptives without exploration of either their abuse history or hormonal status. Depression treatment might vary depending on underlying pathophysiology underscoring the importance of differentiated care. This represents a critical gap in current UK healthcare pathways, where services remain siloed between mental health, DA pathways, and women’s health provision.

Other studies [18, 21] documented increased menopausal symptom reporting among DA survivors, our study reveals an important precursor: the profound uncertainty women experience before reaching the point of symptom identification. In contrast, our participants remained trapped in diagnostic uncertainty, unable to distinguish between trauma responses and hormonal changes. Findings about trauma-menopause relationships [18] are thus complicated by our evidence that many women cannot even reach the point of clear symptom attribution.

The bidirectional relationship between DA and perimenopause emerged as particularly significant. Whilst it is established that trauma histories intensify menopausal symptoms [18], our findings reveal how this creates a vicious cycle: abuse-related stress exacerbates hormonal symptoms, which perpetrators then exploit for further control, which in turn heightens stress and symptoms. This extends understanding beyond simple symptom amplification to reveal a dynamic interplay that perpetrators actively manipulate. The weaponisation of perimenopausal symptoms represents a previously unreported form of coercive control, where women’s biological changes become a tool of abuse. This weaponization has important theoretical implications for understanding coercive control. Whilst existing literature documents how perpetrators exploit women’s mental health, pregnancy, and parenting [29] the exploitation of menopause represents an extension of control into midlife biological transitions. Participants described perpetrators who forced supplements, insisted women were ‘going mad’ and used hormonal symptoms to justify abuse.

A particularly novel finding emerged regarding how perimenopausal experiences influenced survivors’ responses to abuse. Several women described how hormonal changes led to increased clarity about their situations and decreased tolerance for abusive behaviour, sometimes catalysing decisions to leave abusive relationships. Whilst recognising that leaving can be the most dangerous time for DA survivors, this finding suggests that perimenopause might represent a critical window for intervention. The ‘rebirth’ and clarity that participants described challenge deficit-focused narratives about menopause, revealing it as a potential turning point for reclaiming agency. This has important implications for service timing - support services could develop specific outreach for women in midlife transitions.

The barriers to accessing healthcare extend beyond those documented in existing literature on either DA or menopause alone. Survivors described operating in ‘survival mode’, where the immediate demands of managing abuse consumed the psychological resources needed for health-seeking behaviours [30]. Whilst previous work [31, 32] identified barriers to menopause care, our findings reveal an additional layer: the compound effect when trauma responses diminish self-advocacy precisely when navigating dismissive healthcare systems. The women in our study described how abusive partners sometimes interfered with treatment decisions, either through direct control or by creating environments where survivors felt unable to prioritise their health needs. Additionally, the ‘put up and shut up’ mentality described by participants illustrates how sustained abuse erodes the very self-esteem needed to challenge inadequate medical care, creating a perfect storm where those most needing support are least able to advocate for it.

The role of healthcare providers, particularly GPs, emerged as crucial but often problematic. Participants’ experiences ranged from supportive to dismissive, reflecting wider issues in healthcare responses to both DA [33, 34]and menopause [35]. These varied experiences highlight the need for consistent, trauma-informed approaches to supporting women managing both perimenopause and DA experiences.

Financial abuse emerged as a critical but often overlooked barrier to menopause care. Participants described being unable to afford alternative treatments or private healthcare whilst still experiencing economic control from ex-partners - a finding that extends beyond simple poverty to reveal how abusers weaponise financial dependency even post-separation. This intersects with documented disparities in HRT prescribing across socioeconomic groups [5, 36], but adds a crucial dimension: women experiencing DA face both systemic healthcare inequalities and deliberate economic sabotage.

Where formal systems failed, peer support was identified as a valuable support mechanism. Participants consistently valued conversations with other women navigating similar experiences, finding in these informal networks what healthcare providers could not offer: validation that their complex, overlapping symptoms were real and manageable. The Fawcett Society [37] emphasised peer support during menopause, and our findings reveal why this is particularly crucial for DA survivors. Peer networks appear able to provide the trauma-informed understanding that healthcare systems lack. Women who had ‘lived through it’ offered practical strategies for distinguishing trauma responses from hormonal changes, shared experiences of gaslighting, and crucially, believed each other. This suggests that survivor-led peer support fills a critical gap, offering both menopause expertise and trauma awareness in ways that professionalised services currently do not replicate.

The intersection of DA and perimenopause cannot be understood without considering how race, class, and geography compound these experiences. Cultural silences around taboo topics suggested in this study intersect with documented disparities in HRT prescribing, where Black and Asian women receive treatment at rates four to five times lower than white women [5]. When combined with existing inequalities in DA service provision across UK regions [10, 11, 14], women face what amounts to a triple jeopardy: navigating cultural taboos around menopause, systemic healthcare inequalities, and services designed without considering their intersecting needs. Our findings suggest that without explicitly intersectional approaches, support services risk perpetuating these inequalities by failing to recognise how multiple marginalised identities shape women’s experiences of both DA and perimenopause.

Our findings uniquely illuminate how, at the temporal intersection of DA and perimenopause, women’s sense of self and agency is impacted. Unlike studying these experiences separately, we found that the unpredictability of perimenopausal symptoms created new opportunities for gaslighting - when hot flushes came and went, perpetrators insisted women were ‘making it up.’ This temporal confusion had profound effects on participants’ self-perception: some described feeling they were ‘going mad’ when unable to distinguish between trauma responses and hormonal changes. Rather than assuming perimenopause uniformly diminishes capacity, services must recognise it as a potential turning point where women may be simultaneously more vulnerable to manipulation and also more ready for change.

Our findings reveal that the intersection of DA and perimenopause creates a unique form of vulnerability that current support systems are ill-equipped to address. Women navigate not just overlapping symptoms but overlapping silences - the cultural taboos around menopause compounding the isolation of abuse. Yet, within this complexity lies opportunity: participants’ experiences of perimenopause as both weapon and catalyst suggest that midlife transitions, when properly understood and supported, might offer unexpected openings for transformation.

Our findings have direct implications for policy and practice across healthcare, support services, and research priorities. Women described seeking help for symptoms without understanding their origin - one attending the local hospital department thinking she had a heart attack, another fearing Alzheimer’s when experiencing gaslighting. GPs require training to recognise this diagnostic complexity. The scale of this issue demands urgent attention. In England and Wales, over 1 million women sought support as DA survivors in the last 12 months alone [10], with approximately 10% aged between 35 and 54. These women will require menopause support within the next decade, making integrated service development critical.

### Strengths and limitations

This study demonstrates several significant strengths in its approach and execution. The partnership approach to research design prioritised participant safety and wellbeing through the involvement of a trusted community leader as facilitator, creating an environment where DA survivors felt secure in sharing their experiences. The focus group format created an empowering feminist environment of peer support to discuss taboo topics in a safe space. The findings from this study open up new dialogues about the mechanisms of support that women should be afforded, not just the routine questions about DA, but connect the dots between any disclosed abuse and the life-changing effects of perimenopause.

We acknowledge the limitations of the work. The study sample, whilst appropriate for qualitative research, consisted primarily of DA survivors already connected to support services, potentially missing the experiences of women facing additional barriers to accessing help. The online format, though enabling broader geographical participation, may have excluded women without reliable internet access or those experiencing digital abuse [38]. Additionally, whilst the study included some diversity in participants’ backgrounds, it does not fully represent the experiences of women from all cultural and socioeconomic contexts, particularly given documented disparities in accessing both DA and menopause support services. Whilst qualitative small sample work has limits to its generalisability, it can offer useful transferable insights, especially into topics with limited data, such as in this arena of DA and menopause. Menopause research, especially large-scale studies of hormone and biological complexities, should record women’s DA status to enable future examination of the impact of trauma on women’s health experiences. Further qualitative research into the clinical experiences of and perspectives would give useful insights into supporting this group of women.

## Conclusion

This study provides insights into the complex intersection of DA and perimenopause, revealing how these experiences create unique challenges that current support systems fail to address. Our findings demonstrate three interconnected themes: diagnostic uncertainty that leaves women unable to distinguish between trauma responses and hormonal changes; the weaponisation of perimenopausal symptoms as a form of coercive control alongside unexpected empowerment that can catalyse escape; and system failures that drive survivors toward peer networks for the validation and support that professional services cannot provide.

The three interconnected themes reveal how the intersection of DA and perimenopause creates a perfect storm of challenges. The symptom uncertainty described in Theme 1 creates vulnerabilities that can be exploited by abusers (Theme 2), whilst simultaneously making it difficult for survivors to access appropriate support (Theme 3). Yet within this complexity lies potential for transformation - the same hormonal changes that create vulnerability can also catalyse liberation, and where formal systems fail, survivor networks provide crucial lifelines. Understanding these interconnections is essential for developing support services that can respond to the full complexity of women’s lived experiences at this critical life juncture.

Participants sharing reports of perpetrators exploiting perimenopausal symptoms for psychological abuse represents a novel contribution to understanding coercive control in midlife. Equally significant is our finding that perimenopause can serve as a turning point, providing clarity that enables some women to leave abusive relationships. These insights challenge deficit-focused narratives about menopause and highlight the need for services that recognise both the vulnerabilities and strengths of this life transition.

Whilst this qualitative study of 15 women cannot be generalised to all DA survivors experiencing perimenopause, it provides essential insights into an under-researched intersection. The community-partnered approach proved crucial in enabling participants to feel secure and empowered enough to share experiences of both abuse and hormonal transitions, suggesting methodological implications for future research in this sensitive area.

This research underscores the need for integrated, trauma-informed approaches across healthcare and support services. Future research should explore the prevalence of symptom weaponisation, optimal timing for interventions during hormonal transitions, and the development of screening tools that can navigate the situational complexity we have identified. Only through recognising the full complexity of women’s life course can services provide the integrated support that survivors need and deserve.

## Supplementary Information

Supplementary Material 1.

## Data Availability

The datasets generated and/or analysed during the current study are not publicly available due to the personal and potentially identifiable nature of the qualitative data. Participants provided consent for anonymised use within the context of local research only. Data are not available for sharing beyond what is included in this published article.

## Abbreviations

A&E: Accident and Emergency
AI: Artificial Intelligence
CPTSD: Complex Post-Traumatic Stress Disorder
CSEW: Crime Survey of England and Wales
DA: Domestic Abuse
HRT: Hormone Replacement Therapy
OSOP: One Sheet of Paper
UK: United Kingdom
