# Supplementary material for: ‘They can induce and exacerbate each other’ – the complex interplay between domestic abuse and the perimenopause: a qualitative study with female survivors
Source: BMC Womens Health. 2025 Nov 29;26:7. doi: 10.1186/s12905-025-04080-9 (PMC12772039; doi:10.1186/s12905-025-04080-9)
Supplement: Supplementary file 1 — Supplementary Material 1. [file 12905_2025_4080_MOESM1_ESM.docx]

**Supplementary Data S1**

Interview Schedule (Co-designed with community partners)

| **Semi-structured interview questions** (with prompts)  **Q1) Tell me about your menopause experience**   - Where are you in your menopause journey? - When did your menopause journey start? - How/When did you know it was the menopause? - What symptoms have you experienced? - How has it been for you so far?   **Q2) Tell me about accessing menopause support and information**   - How do you think YOUR experience of the menopause might be different to others? - Where do you access support and information about the menopause?   - How do you decide if you can trust information online?   - Do you think there should be more/better access to support and information about the menopause for women like you? What might this look like – any suggestions?   - What do you think of / about menopause ‘role models’?   **Q3) Your community and future work**   - What are the barriers to accessing care and support for women in your community? - What would help women in your community access care and support through the menopause? - What kind of support would your community value most for the menopause? |
| --- |
